# Novel variants in LSS related hypotrichosis simplex 14

**DOI:** 10.3389/fgene.2026.1742964

**Published:** 2026-06-17

**Authors:** Shengyuan Hua, Biqing Fang, Yile Cheng, Xinhui Zhang, Yulin Zhang, Qiufang Qian, Jia Zhang

**Affiliations:** 1 Department of Dermatology, Shanghai Children’s Hospital, School of Medicine, Shanghai JiaoTong University, Shanghai, China; 2 Department of Dermatology, Shanghai City Qingpu District of Zhujiajiao People’s Hospital, Shanghai, China; 3 Shanghai Municipal Hospital of Traditional Chinese Medicine, Shanghai University of Traditional Chinese Medicine, Shanghai, China; 4 Department of Dermatology, QuanZhou Women’s and Children’s Hospital, Quanzhou, China; 5 Department of Dermatology, Yueyang Hospital of Integrative Chinese and Western Medicine, Shanghai University of Traditional Chinese Medicine, Shanghai, China; 6 Department of Dermatology, Xinhua Hospital Affiliated to Shanghai Jiaotong University School of Medicine, Shanghai, China

**Keywords:** Chinese, hypotrichosis simplex, LSS, novel variants, teeth dysplasia

## Abstract

**Background:**

Hypotrichosis simplex (HS) is a rare form of hereditary alopecia caused by a variety of gene variants, with onset in childhood. Few studies regarding *LSS*-related HS(HS 14) have been reported and genotype–phenotype correlations in the *LSS* gene are still not completely clear.

**Methods:**

In this study, we tried to make a definite diagnosis in two Chinese pediatric patients clinically suspected of congenital hypotrichosis. Peripheral blood samples of these two patients and their parents were collected and whole-exome sequencing (WES) was performed to elucidate the genetic cause. WES revealed four different compound heterozygous variants in *LSS* in two probands that confirmed a diagnosis HS 14, including two novel variants. AlphaFold two was performed to predict three-dimensional structures, and the PROVEAN analysis software was utilized to assess the functional changes of novel missense variant.

**Results:**

Two previously reported variants,c.1054G>A; p.(Gly352Arg) and c.1303C>T; p.(Arg435Cys) were observed and two novel heterozygous variants,c.1594G>C; p.(Glu532Gln) and c.1010C>T; p.(Pro337Leu) were found in these 2 HS patients.

**Conclusion:**

In this study, we successfully identified variants in *LSS* in two probands that confirmed a diagnosis of HS, including two novel variants. These findings expanded the variantal spectrum of *LSS*-related HS 14. Moreover, teeth dysplasia could be an associated phenotype in patient with HS 14.

## Introduction

Hypotrichosis simplex type 14(HS 14) is a rare autosomal recessive disorder characterized by hair loss abnormality, typically manifesting in infancy or early childhood causing significant psychological impact on patients (Linfang et al., 2024). It is characterized by sparse, soft, lanugo-like hair, even no hair, sparse and fragile eyebrows, eyelashes and body hair. The *LSS* gene (located on chromosome 22q1.3 and containing 23 exons) encodes lanosterol synthase, an enzyme that plays a key role in cholesterol metabolism, particularly in hair follicle cells. In recent years, few studies have shown that the variant of *LSS* gene is the main cause of HS 14 (Linfang et al., 2024). In this study, we successfully identified variants in *LSS* in two probands that confirmed a diagnosis of HS 14, including two novel variants. These findings expanded the mutational spectrum and revealed that teeth dysplasia observed in one proband could be an associated phenotype of *LSS-*related HS 14.

## Case report

**FIGURE 1:**
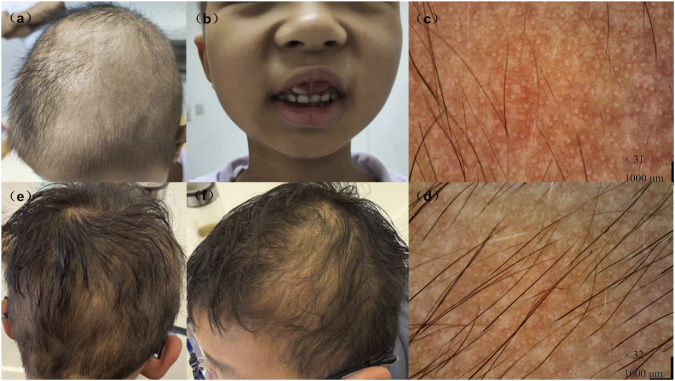
**(a,b)** Clinical pictures of proband one:including HS and dental dysplasia. **(c,d)** Dermoscopy performance of proband 1: There were white halos around some hair follicles. **(e,f)** Clinical pictures of proband 2: Sparse and thin hair had been present since infancy.

Proband 1,a 6-year-old girl, exhibited recurrent hair loss after birth, aggravated in summer, mild in winter, which persisted through early childhood (Figure 1a). Trichoscopy examination showed that most of the pulled hairs were in anagen phase with a few twisted hairs visible. There were white halos around some hair follicles (Figures 1c,d). Her baby teeth did not fall out, and permanent teeth eruptted above the gums (Figure 1b). Her elder brother also have the same symptom but both parents have no history of baldness. Both children had no abnormalities in eyebrows, nails, vision and intelligence. Pedigree can be referred to in Supplementary Figure 1a.

The proband two was a 6-year-old boy and he was the second son of a healthy couple. Sparse and thin hair had been present since infancy (Figures 1e,f), but without pain, pruritus. Physical examination revealed no deformity of the limbs, teeth and no intellectual disability or congenital cataract. Pedigree can be referred to in Supplementary Figure 1b.

This study received approval from the Institutional Review Committee of Xinhua Hospital Affiliated to Shanghai Jiaotong University School of Medicine, and was carried out in compliance with the principles of the Declaration of Helsinki. Following the procurement of written informed consent from all participants, peripheral blood samples were collected for the purposes of DNA extraction and genetic testing.

Whole-exome sequencing(WES) as previously reported (Khan et al., 2022) was conducted on two families to detect variants in all affected individuals and their unaffected family members. Exome capture was performed using the Agilent SureSelect Human All Exon Kits (Agilent, Santa Clara, CA, USA), following the manufacturer’s protocols. Sequencing was carried out on a HiSeq 2000 platform with paired-end reads of 100 bp, consistent with our previously reported methodology (Li. et al., 2015). Sanger sequencing was subsequently employed to validate the candidate variant identified through WES.

Sequencing results revealed four distinct compound heterozygous variant in the *LSS* gene. Variant c.1054G>A; p.(Gly352Arg) and c.1594G > C; p.(Glu532Gln) in proband 1,while variant c.1303C>T; p.(Arg435Cys) and c.1010C>T; p.(Pro337Leu) in proband 2 (Supplementary Figure 2), two of which(c.1594G>C; p.(Glu532Gln) and c.1010C>T; p.(Pro337Leu)have not been reported in published work or online databases, including the 1000 Genomes Project, HapMap8 and dbSNP135 (variant in the *LSS* gene is described with the reported cDNA reference sequence, GenBank accession no. NM_002340.5).

Online *in silico* programs PolyPhen2 (http://genetics.b wh. harvard.edu/pph2) and SIFT (http://sift.bii.a-star.edu.sg) were applied to predict the impact of novel missense variant. Variants c.1010C>T; p.(Pro337Leu) and c.1594G>C; p.(Glu532Gln) were all predicted to be “possibly damaging” with Polyphen2 and “deleterious” with SIFT. According to the ACMG guidelines, the variant c.1594G>C (p.Glu532Gln) is classified as Likely Pathogenic(class 4), with supporting evidence: PM1 + PM2_Supporting + PM3 + PP3.

For the variant c.1010C>T (p.Pro337Leu), its pathogenicity is classified as Uncertain Significance(class 3) based on the ACMG criteria. The supporting evidence includes:

PM2_Supporting: The allele frequency in general population databases is 0.000077.

PP3: Multiple *in silico* functional prediction tools, including REVEL, suggest a likely deleterious effect on protein function.

The PROVEAN (Yongwook and Agnes, 2015) analysis software was utilized to assess the functional changes in the protein resulting from these two variants. The UniProt ID of LSS is P48449. To investigate the potential functional consequences of the c.1010C>T; p.(Pro337Leu) and c.1594(exon17)G > C; p.(Glu532Gln) variants, we first predicted their 3D structures using AlphaFold2 (John et al., 2021). Although a protein structure for LSS exists in the PDB database, no structural information is available for the mutated residues Pro337Leu and Glu532Gln. The wild-type and mutant LSS structures were refined based on the predicted protein models. Structural models were generated using PyMOL software (www.pymol.org).

**FIGURE 2:**
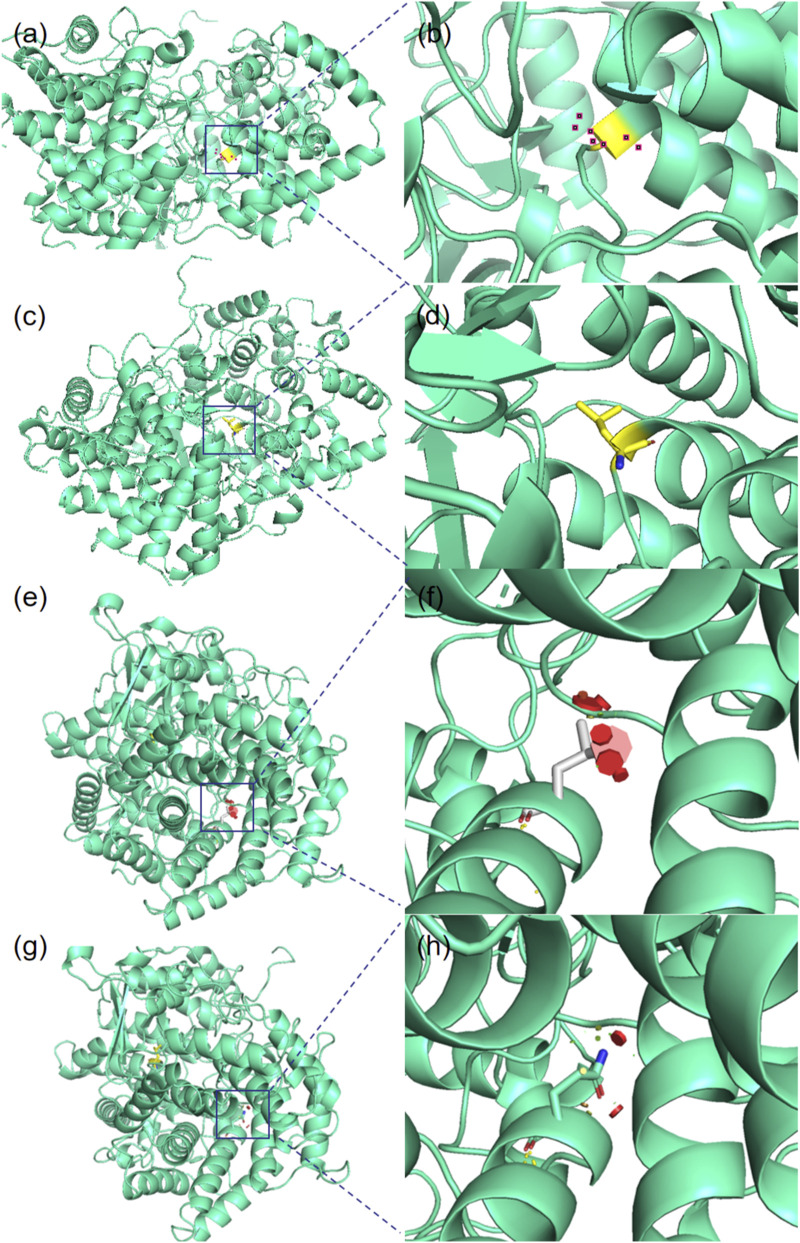
**(a,c)** 3D Structure of the Wild-Type Protein. **(b)** Enlarged View of Wild-Type Protein: Residue P377 is highlighted in yellow. **(d)** Mutant LSS (P377L): The mutated residue L377 is shown in yellow. This variant may affect protein structure and function. **(e,g)** 3D Structure of the Wild-Type Protein. **(f)** Enlarged View of Wild-Type Protein: Residue E532 is highlighted in white. **(h)** Mutant LSS (E532Q): The mutated residue Q532 is shown in white. This variant may affect protein structure and function.

According to our analysis, both variants significantly impair the stability and functionality of the LSS protein. Their predicted effects are ranked in descending order of PROVEAN scores as follows: p. Pro337Leu (−9.813) > p. Glu532Gln (−2.897), which is below the threshold of −2.5, suggesting a deleterious effect. As shown in Figure 2, both mutated amino acid residues are located within the interior of the protein. Specifically, the p. Pro337Leu variant substitutes Proline with Leucine (Figures 2b,d). The mutation has significant detrimental to the LSS protein and markedly reduces its stability, transforming a basic amino acid into a polar one. While the p. Glu532Gln variant replaces Glutamic acid with Glutamine (Figures 2f,h). The mutation could compromise the three-dimensional stability of the Glu532Gln protein thereby substantially altering the charge distribution of LSS.

## Discussion

The phenotype of our probands clinically aligns *LSS*-related HS: early-onset, progressive hair loss without associated neurological deficits. In this study, we identified two novel compound heterozygous variants, c.1010C>T; p.(Pro337Leu) and c.1594G>C; p.(Glu532Gln), in the LSS gene from two unrelated Chinese families with autosomal recessive HS14. Through a comprehensive comparison with all previously reported LSS variants summarized in Table 1, we confirmed that these two variants have not been documented in public databases (including 1,000 Genomes, gnomAD, and dbSNP) or existing literatures, thus expanding the known mutation spectrum of the LSS gene. Notably, the other two variants detected in our patients, c.1054G>A; p.(Gly352Arg) and c.1303C>T; p.(Arg435Cys), have been previously reported in Chinese HS14 cohorts. Consistent with Table 1, c.1303C>T (p.Arg435Cys) was specifically linked to hair–tooth ectodermal dysplasia (Shengyuan et al., 2020; Bao et al., 2025), whereas c.1054G>A (p.Gly352Arg) represented a recurrent variant in Chinese HS14 patients. The presence of this variant in our Proband 1, who also exhibited dental abnormalities, further validates the genotype-phenotype correlation between p. Arg435Cys and ectodermal dysplasia involving both hair and teeth. This consistency strengthens the notion that p. Arg435Cys represents a key variant contributing to syndromic features beyond isolated hypotrichosis. The structural analysis of these variants offers evidence of pathogenicity.

**TABLE 1:** Review of published work: patients with LSS mutations.

No.	Nt change	AA change	Phenotype	References
1	c.1547A>G	p.Asn516Ser	Developmental delay, neurological abnormality, alopecia	Besnard et al. (2019)
2	c.2114C>A c.779G>C	p.Thr705Lys p.Arg260Pro	Developmental delay, neurological abnormality, alopecia	Besnard et al. (2019)
3	c.1194 + 5G>A c.1109 + 2T>C	splice p.? splice p.?	Developmental delay, neurological abnormality, alopecia	Besnard et al. (2019)
4	c.1109 + 2T>C c.857A>G	splice p.? p.Tyr286Cys	Developmental delay, neurological abnormality, alopecia	Besnard et al. (2019)
5	c.1810C>T c.1417dup	p.Arg604* p.His473Profs*32	Developmental delay, neurological abnormality, alopecia	Besnard et al. (2019)
6	c.41A>G Unidentified but allelic imbalance	p.Tyr14Cys	Developmental delay, neurological abnormality, alopecia	Besnard et al. (2019)
7	c.1955C>T c.35G>A	p.Thr652Ile p.Gly12Asp	Developmental delay, neurological abnormality, alopecia	Besnard et al. (2019)
8	c.35G>A c.1741T>C	p.Gly12Asp p.Trp581Arg	Congenital cataract	Zhao et al. (2015)
9	c.1762G>A c.1025T>C	p.Gly588Ser p.Ile342Ser	Congenital cataract, baldness, absence of eyebrows and a small penis	Chen and Liu (2017)
10	c.1887G>T c.1054G>A	p.Trp629Cys p.Gly352Arg	Sparse and thin hair	Li et al. (2019)
11	c.1885T>A c.1172T>C(hom)	p.Trp629Arg p.Phe391Ser(hom)	Sparse scalp hair and a pronounced paucity of body hair	Romano et al. (2018)
12	c.625A>T c.423G>A	p.Asn209Tyr p.Trp141*	Congenital alopecia; sparse, lanugo-like scalp hair, sparse and brittle eyebrows, sparse and thinned eyelashes, sparse hair on the extremities; absence of axillary and pubic hair	Romano et al. (2018)
13	c.304C>G c.743T>C	p.Leu102Val p.Leu248Pro	Sparse, lanugo-like scalp hair, sparse and brittle eyebrows, normal eyelashes, normal hair on the extremities, sparse pubic hair and an absence of axillary hair	Romano et al. (2018)
14	c.530G>A c.701_716del	p.Arg177Gln p.Arg234Profs2*	Congenital hypotrichosis, midline anomalies, such as cleft palate and agenesis of the corpus callosum, and no cataracts	Wada et al. (2020)
15	c.1196C>A c.706G>A	p.Ala399Glu p.Val236Met	Sparse and thin hair, sparse and brittle eyebrows, sparse and thinned eyelashes	Hua et al. (2020)
16	c.1303C>T c.1887G>T	p.Arg435Cys p.Trp629Cys	Sparse and thin hair, sparse and brittle eyebrows, sparse and thinned eyelashes, teeth dysplasia	Hua et al. (2020)
17	c.812T>C	p.Ile271Thr	Scalp hair: sparse and thin; hair on the limbs, axillary regions and pubic regions: absent; atrophy of the right kidney, azoospermia, hypergonadotropic hypogonadism	Zhang et al. (2025)
18	c.919_921del c.812T>C	p.His307del p.Ile271Thr	Scalp hair: sparse vellus hair; periungual erythema, scaling, and hyperkeratosis on all fingers	Zhang et al. (2025)
19	c.1025T>G c.934 C>T	p.Ile342Ser p.Arg312Trp	Scalp hair, eyebrows and eyelashes: almost absent; cataract	Zhang et al. (2025)
20	c.1987 C>T c.982 C>T	p.Arg663Trp p.Arg328	Scalp hair: sparse; scalp folliculitis, itchiness	Zhang et al. (2025)
21	c.1405_1407del c.193_200dup	p.Glu469del p.Pro68Argfs*14	Scalp hair: thin and weak, sparse Over the entire scalp	Zhang et al. (2025)
22	c.1405_1407del c.193_200dup	p.Glu469del p.Pro68Argfs*14	Scalp hair: sparse	Zhang et al. (2025)
23	c.530 G>A	p.Arg177Gln	Sparse hair, eyelashes and eyebrows were unremarkable, developmental speech disorder, learning difficulties, and microcephaly	Cesarato et al. (2021)
24	c.934 C>T c.881 G>T	p.Arg312Trp p.Arg294Leu	Scalp hair, sparse and very light colored eyelashes and eyebrows, hearing difficulties, concentration problems	Cesarato et al. (2021)
25	c.1702 C> T	p.Arg568 Trp	Sparse scalp hair from birth	Cesarato et al. (2021)
26	c.393 G > A	p.131Leu=	Scant fluffy hair on the scalp, missing eyebrows and body hair, and very sparse eyelashes	Cesarato et al. (2021)
27	c.530 G> A c.1460 T>A	p.R177Q p.V487E	Short and thin vellus hairs on their scalp and were easily plucked	Murata et al. (2020)
28	c.711 C>G c.1646 C>T	p.Y237 p.P549L	Short and thin vellus hairs on their scalp and were easily plucked	Murata et al. (2020)
29	c.812 T>C	p.Ile271Thr	Thin scalp hair and normal eyebrows	Zhao et al. (2023)
30	c.1609 G>T	p.Val537Leu	Congenital alopecia, the complete absence of eyelashes and eyebrows, and global developmental delay	Elaraby et al. (2023)
31	c.14 + 2 T>C c.1357 G>A	p?; p.Val453Ile	Alopecia with intellectual disability, growth retardation, agenesis of corpus callosum, hypogenitalism	Elaraby et al. (2023)
32	c.818 G>A c.1025 T>G	p.Trp273Ter p.Ile342Ser	Cataract, hypotrichosis, palmoplantar keratoderma	Ho et al. (2022)
33	c.3 G>A c.1025 T>G	p.Met1? p.Ile342Ser	Palmoplantar keratoderma with Alopecia, cataract, pseudoainhum, agenesis of corpus callosum	Yang et al. (2022)
34	c.1522 G>T c.428 + 42 T>A	p.Gly508Trp p.?	Palmoplantar keratoderma with Alopecia, cataract, pseudoainhum, agenesis of corpus callosum	Yang et al. (2022)
35	c.683 C>T c.779 G>A	p.Thr228Ile p.Arg260His	Palmoplantar keratoderma	Zhou et al. (2023)
36	c.1303 C>T c.386 G>A	p.Arg435Cys p.Arg129Gln	Congenital hypotrichosis and intermittent exotropia, sparse hair with yellow color, reduced strength, and minimal growth	Bao et al. (2025)
37	c.968T> C c.1799G>T	p.Ile323Thr p.Gly600Val	Alopecia on her scalp and eyebrows since birth	El Hakim et al. (2023)
38	c.1030A>G c.1509T>G	p.Met344Val p.Tyr503	Sparse slowly growing hair from birth	Morice-Picard et al. (2023)
39	c.1054 G>A c.1594 G>C	p. Gly352Arg p. Glu532Gln	Hair loss after birth	This report
40	c.1303 C>T c.1010 C>T	p. Arg435Cys p. Pro337Leu	Sparse and thin hair	This report

Trichoscopic findings in Proband one further supported the diagnosis of LSS-related hypotrichosis simplex. Dermoscopy showed that most plucked hairs were in the anagen phase, with occasional twisted hairs observed. In addition, perifollicular white halos were seen around some hair follicles. These features are consistent with impaired hair follicle structure and hair shaft abnormalities caused by cholesterol metabolism disturbance in LSS deficiency. The perifollicular white halos may reflect mild inflammation, fibrosis, or abnormal keratinization around the hair follicle, which has rarely been described in LSS-related hypotrichosis. These trichoscopic signs can serve as auxiliary clues for clinical screening and differential diagnosis before genetic testing is performed.

The core of the discussion lies in the functional implications of the novel variants. Lanosterol synthase is a key enzyme in the cholesterol biosynthesis pathway, catalyzing the cyclization of oxidosqualene to lanosterol. Cholesterol and its derivatives, such as steroid hormones and bile acids, are critical for hair follicle cycling, signaling, and structural integrity. The p. Pro337Leu and p. Glu532Gln variants, both predicted to be deleterious, are located within the protein’s interior. The substitution of Proline-337 with Leucine is particularly disruptive. Proline imposes a rigid kink in the protein backbone, and its replacement with a flexible Leucine residue likely causes significant local conformational strain, potentially misfolding the protein or impairing its catalytic cavity. This is corroborated by its profoundly negative PROVEAN score (−9.813), indicating a severe impact. Similarly, the p. Glu532Gln variant replaces a negatively charged, acidic residue with a neutral, polar one. This alteration in charge distribution could destabilize crucial salt bridges or hydrogen-bonding networks within the protein’s core, compromising its stability. Our 3D modeling supports this, showing both mutant residues buried in structurally critical regions. Consequently, these variants are predicted to lead to a loss-of-function of the LSS enzyme, disrupting the local cholesterol synthesis in the hair follicle. This metabolic deficit is thought to impair the signaling and structural processes essential for anchoring the hair shaft and maintaining the hair cycle, ultimately leading to the production of weak, easily shedded hair.

A particularly intriguing finding is the association of the compound heterozygous variant; p.(Arg435Cys and p. Glu532Gln) with dental dysplasia in proband 1,.In our previously published article, we have also reported a case of HS patients with a phenotype of hypotrichosis simplex and sparse teeth caused by *LSS* variant (Shengyuan et al., 2020). Variant c.1303C>T; p.(Arg435Cys) and c.1887G>T; p.(Trp629Cys) were reported in that case, which makes it hard for us not to doubt whether the co-occurring gene variant c.1303C>T; p.(Arg435Cys) is the cause or key to dental dysplasia phenotype. Although *LSS* variants are mainly associated with isolated hypotrichosis, emerging evidence indicates that it has a broader phenotypic spectrum, including palmoplantar keratoderma (OMIM # 144200) (Bao et al., 2025),congenital cataract-44 (OMIM # 616509) (Ling et al., 2015),and alopecia intellectual disability syndrome 4 (APMR4, OMIM # 618840) (Thomas et al., 2019),which suggests that specific *LSS* alleles, particularly those inducing more profound structural destabilization, may confer a risk for non-follicular manifestations.

In conclusion, our study provides supportive genetic evidence for the diagnosis of HS14 in two families and identifies two novel LSS variants. We propose that the severity and nature of the structural disruption caused by a variant influences the phenotypic outcome. The association of the compound heterozygous variant; p.(Arg435Cys and p. Glu532Gln) with dental dysplasia highlights the need for comprehensive clinical evaluations in HS14 patients, moving beyond the scalp to include oral and other ectodermal examinations. Future functional studies, such as enzymatic assays on mutant proteins, are essential to definitively confirm the pathogenic impact of these variants and to further unravel the intricate link between cholesterol metabolism and human morphogenesis.

## Data Availability

The original contributions presented in the study are included in the article/Supplementary Material, further inquiries can be directed to the corresponding authors.

## Supplementary material

SUPPLEMENTARY FIGURE S1 Pedigrees of the two families with the genetic disorder. **(a)** Family 1. **(b)** Family 2. The arrow indicates the proband. Squares represent males, circles represent females. Filled symbols indicate affected individuals, half-filled symbols indicate heterozygous carriers, and open symbols indicate unaffected individuals.

SUPPLEMENTARY FIGURE S2 In proband 1, the variants c.1054G>A(p.Gly352Arg) and c.1594G>C(p.Glu532Gln) in LSS were revealed in a compound heterozygous state. **(c,d)** Two heterozygous variants c.1303C>T(p.Arg435Cys) and c.1010C>T(p.Pro337Leu) were identified in proband 2.
