# Supplementary figures and images for: Novel variants in LSS related hypotrichosis simplex 14
Source: Front Genet. 2026 Jun 17;17:1742964. doi: 10.3389/fgene.2026.1742964 (PMC13318249; doi:10.3389/fgene.2026.1742964)

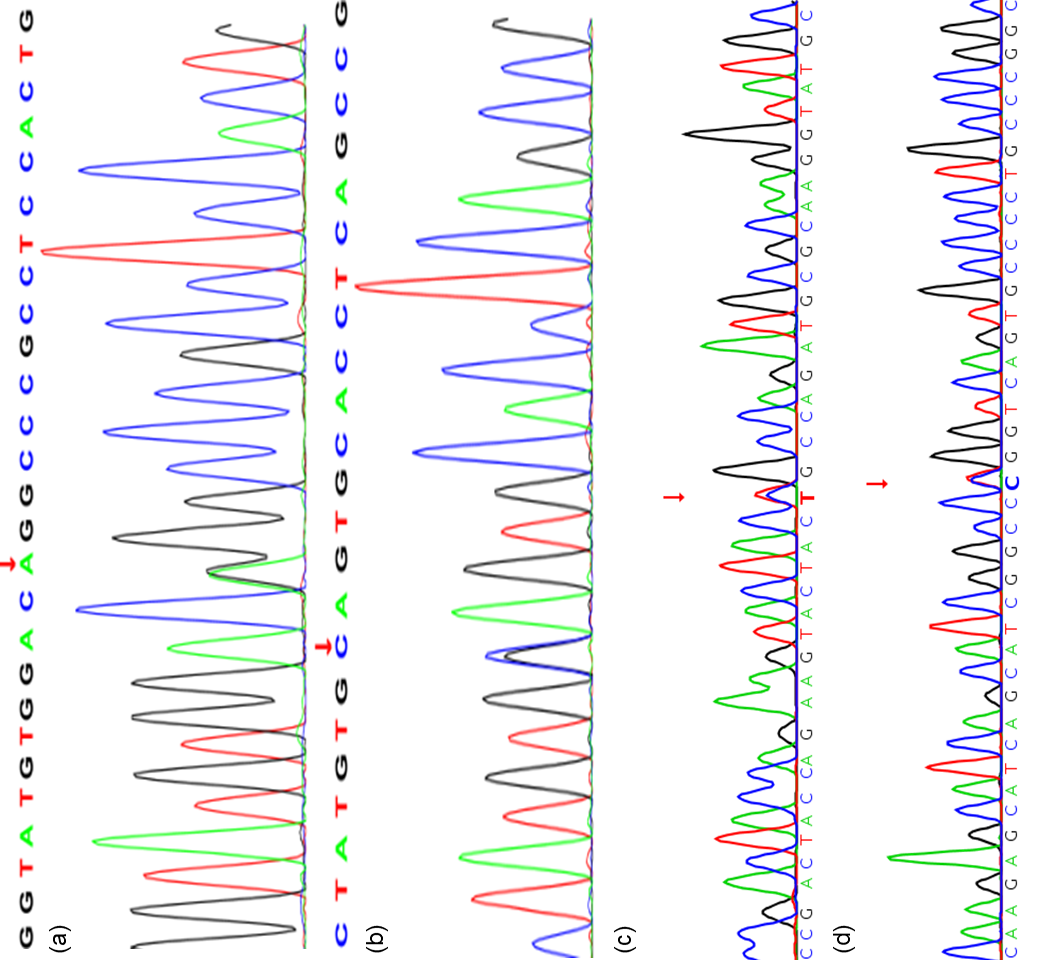

Supplement: Supplementary file 1 [file Image2.TIF]

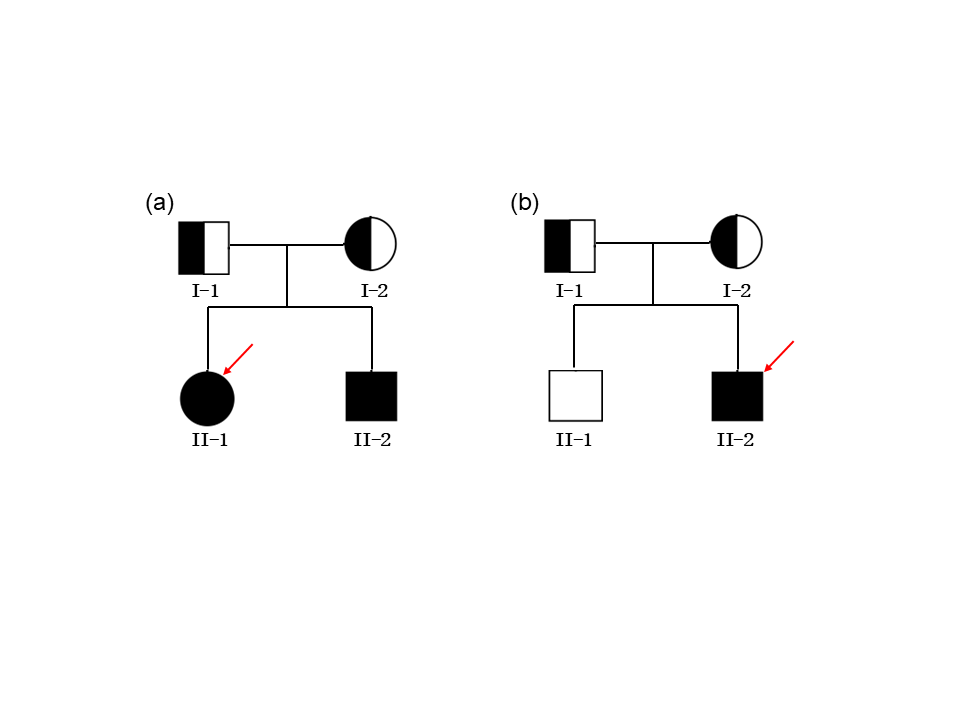

Supplement: Supplementary file 2 [file Image1.TIF]
