# Supplementary material for: Novel variants in LSS related hypotrichosis simplex 14
Source: Front Genet. 2026 Jun 17;17:1742964. doi: 10.3389/fgene.2026.1742964 (PMC13318249; doi:10.3389/fgene.2026.1742964)
Supplement: Supplementary file 3 [file Table1.doc]

| **No.** | **Nt change** | **AA change** | **Phenotype** | **References** |
| --- | --- | --- | --- | --- |
| 1 | c.1547A>G | p.Asn516Ser | Developmental delay, neurological abnormality, alopecia | Besnard et al., 2019 |
| 2 | c.2114C>A  c.779G>C | p.Thr705Lys  p.Arg260Pro | Developmental delay, neurological abnormality, alopecia | Besnard et al., 2019 |
| 3 | c.1194 + 5G>A  c.1109+2T>C | splice p.?  splice p.? | Developmental delay, neurological abnormality, alopecia | Besnard et al., 2019 |
| 4 | c.1109+2T>C  c.857A>G | splice p.?  p.Tyr286Cys | Developmental delay, neurological abnormality, alopecia | Besnard et al., 2019 |
| 5 | c.1810C>T  c.1417dup | p.Arg604*  p.His473Profs*32 | Developmental delay, neurological abnormality, alopecia | Besnard et al., 2019 |
| 6 | c.41A>G  Unidentified but allelic imbalance | p.Tyr14Cys | Developmental delay, neurological abnormality, alopecia | Besnard et al., 2019 |
| 7 | c.1955C>T  c.35G>A | p.Thr652Ile  p.Gly12Asp | Developmental delay, neurological abnormality, alopecia | Besnard et al., 2019 |
| 8 | c.35G>A  c.1741T>C | p.Gly12Asp  p.Trp581Arg | Congenital cataract | Zhao et al., 2015 |
| 9 | c.1762G>A  c.1025T>C | p.Gly588Ser  p.Ile342Ser | Congenital cataract, baldness, absence of eyebrows and a small penis | Chen et al., 2017 |
| 10 | c.1887G>T  c.1054G>A | p.Trp629Cys  p.Gly352Arg | Sparse and thin hair | Li et al., 2019 |
| 11 | c.1885T>A  c.1172T>C(hom) | p.Trp629Arg  p.Phe391Ser(hom) | Sparse scalp hair and a pronounced paucity of body hair | Romano et al., 2018 |
| 12 | c.625A>T  c.423G>A | p.Asn209Tyr  p.Trp141* | Congenital alopecia; sparse, lanugo-like scalp hair, sparse and brittle eyebrows, sparse and thinned eyelashes, sparse hair on the extremities; absence of axillary and pubic hair | Romano et al., 2018 |
| 13 | c.304C>G  c.743T>C | p.Leu102Val  p.Leu248Pro | Sparse, lanugo-like scalp hair, sparse and brittle eyebrows, normal eyelashes, normal hair on the extremities, sparse pubic hair and an absence of axillary hair | Romano et al., 2018 |
| 14 | c.530G>A  c.701_716del | p.Arg177Gln  p.Arg234Profs2* | Congenital hypotrichosis, midline anomalies, such as cleft palate and agenesis of the corpus callosum, and no cataracts | Wada et al., 2020 |
| 15 | c.1196C>A  c.706G>A | p.Ala399Glu  p.Val236Met | Sparse and thin hair, sparse and brittle eyebrows, sparse and thinned eyelashes | Shengyuan et al., 2020 |
| 16 | c.1303C>T  c.1887G>T | p.Arg435Cys  p.Trp629Cys | Sparse and thin hair, sparse and brittle eyebrows, sparse and thinned eyelashes, teeth dysplasia | Shengyuan et al., 2020 |
| 17 | c.812T>C | p.Ile271Thr | scalp hair: sparse and thin; hair on the limbs, axillary regions and pubic regions: absent; atrophy of the right kidney, azoospermia, hypergonadotropic hypogonadism | Yujing Zhang et al., 2025 |
| 18 | c.919_921del  c.812T>C | p.His307del  p.Ile271Thr | scalp hair: sparse vellus hair; periungual erythema, scaling, and hyperkeratosis on all fingers | Yujing Zhang et al., 2025 |
| 19 | c.1025T>G  c.934 C>T | p.Ile342Ser  p.Arg312Trp | scalp hair, eyebrows and eyelashes: almost absent; cataract | Yujing Zhang et al., 2025 |
| 20 | c.1987 C>T  c.982 C>T | p.Arg663Trp  p.Arg328 | scalp hair: sparse; scalp folliculitis, itchiness | Yujing Zhang et al., 2025 |
| 21 | c.1405_1407del  c.193_200dup | p.Glu469del  p.Pro68Argfs*14 | scalp hair: thin and weak, sparse  over the entire scalp | Yujing Zhang et al., 2025 |
| 22 | c.1405_1407del  c.193_200dup | p.Glu469del  p.Pro68Argfs*14 | scalp hair: sparse | Yujing Zhang et al., 2025 |
| 23 | c.530 G>A | p.Arg177Gln | sparse hair, eyelashes and eyebrows were unremarkable, developmental speech disorder, learning difficulties, and microcephaly | Nicole Cesarato et al., 2021 |
| 24 | c.934 C>T  c.881 G>T | p.Arg312Trp  p.Arg294Leu | scalp hair, sparse and very light colored eyelashes and eyebrows, hearing difficulties, concentration problems | Nicole Cesarato et al., 2021 |
| 25 | c.1702 C> T | p.Arg568 Trp | sparse scalp hair from birth | Nicole Cesarato et al., 2021 |
| 26 | c.393 G > A | p.131Leu= | scant fluffy hair on the scalp,missing eyebrows and body hair, and very sparse eyelashes | Nicole Cesarato et al., 2021 |
| 27 | c.530 G> A  c.1460 T>A | p.R177Q  p.V487E | short and thin vellus hairs on their scalp and were easily plucked | Mami MURATA et al., 2020 |
| 28 | c.711 C>G  c.1646 C>T | p.Y237  p.P549L | short and thin vellus hairs on their scalp and were easily plucked | Mami MURATA et al., 2020 |
| 29 | c.812 T>C | p.Ile271Thr | thin scalp hair and normal eyebrows | Bei Zhao et al., 2022 |
| 30 | c.1609 G>T | p.Val537Leu | congenital alopecia, the complete absence of eyelashes and eyebrows, and global developmental delay | Nesma M. Elaraby et al., 2022 |
| 31 | c.14+2 T>C  c.1357 G>A | p?;p.Val453Ile | Alopecia with intellectual disability, growth retardation, agenesis of corpus callosum, hypogenitalism | Hasnaa M. Elbendary et al., 2023 |
| 32 | c.818 G>A  c.1025 T>G | p.Trp273Ter  p.Ile342Ser | Cataract, hypotrichosis, palmoplantar keratoderma | S Ho et al., 2022 |
| 33 | c.3 G>A  c.1025 T>G | p.Met1?  p.Ile342Ser | palmoplantar keratoderma with Alopecia, Cataract,  pseudoainhum, agenesis of corpus callosum | Yang et al., 2022 |
| 34 | c.1522 G>T  c.428+42 T>A | p.Gly508Trp  p.? | palmoplantar keratoderma with Alopecia, Cataract,  pseudoainhum, agenesis of corpus callosum | Yang et al., 2022 |
| 35 | c.683 C>T  c.779 G>A | p.Thr228Ile  p.Arg260His | palmoplantar keratoderma | Zhou et al., 2023 |
| 36 | c.1303 C>T  c.386 G>A | p.Arg435Cys  p.Arg129Gln | congenital hypotrichosis and intermittent exotropia, sparse hair with yellow color, reduced strength, and minimal growth | Linlin Bao et al., 2025 |
| 37 | c.968T> C  c.1799G>T | p.Ile323Thr  p.Gly600Val | alopecia on her scalp and eyebrows since birth | Joelle El Hakim et al., 2023 |
| 38 | c.1030A>G  c.1509T>G | p.Met344Val  p.Tyr503 | sparse slowly growing hair from birth | Fanny Morice-Picard et al., 2023 |
| 39 | c.1054 G>A  c.1594 G>C | p. Gly352Arg  p. Glu532Gln | hair loss after birth | This report |
| 40 | c.1303 C>T  c.1010 C>T | p. Arg435Cys  p. Pro337Leu | Sparse and thin hair | This report |

1 Maria-Teresa, Romano., Aylar, Tafazzoli., Maximilian, Mattern., Sugirthan, Sivalingam., Sabrina, Wolf., Alexander, Rupp., et al.(2018). Bi-allelic Mutations in LSS, Encoding Lanosterol Synthase, Cause Autosomal-Recessive Hypotrichosis Simplex. *Am. J. Hum. Genet.* 103(5), 777-785. doi:10.1016/j.ajhg.2018.09.011

2 Xiaodan, Chen.,and Li, Liu.(2017). Congenital cataract with LSS gene mutations: a new case report. *J. Pediatr. Endocrinol. Metab.* 30(11), 1231-1235. doi:10.1515/jpem-2017-0101

3 Thomas, Besnard., Natacha, Sloboda., Alice, Goldenberg., Sébastien, Küry., Benjamin, Cogné., Flora, Breheret., et al. (2019). Biallelic pathogenic variants in the lanosterol synthase gene LSS involved in the cholesterol biosynthesis cause alopecia with intellectual disability, a rare recessive neuroectodermal syndrome. *Genet. Med.* 21(9), 2025-2035. doi:10.1038/s41436-019-0445-x

4 Fucheng, Li., Can, Liao., Ru, Li., Yongling, Zhang., Xiangyi, Jing., Yan, Li., et al.(2019). A novel and a known mutation in LSS gene associated with hypotrichosis 14 in a Chinese family. *J. Dermatol.* 46(11), e393-e395. doi:10.1111/1346-8138.15010

5 Yoichi, Wada., Atsuo, Kikuchi., Akimune, Kaga., Naoki, Shimizu., Junya, Ito., Ryo, Onuma., et al.(2020). Metabolic and pathologic profiles of human LSS deficiency recapitulated in mice. *PLoS Genet.* 16(2), e1008628. doi:10.1371/journal.pgen.1008628

6 Ling, Zhao., Xiang-Jun, Chen., Jie, Zhu., Yi-Bo, Xi., Xu, Yang., Li-Dan, Hu., et al.(2015). Lanosterol reverses protein aggregation in cataracts. *Nature* 523(7562), 607-11. doi:10.1038/nature14650

7 Shengyuan, Hua., Yu, Ding., Jia, Zhang., Qiufang, Qian., and Ming, Li.(2020). Novel mutations in Chinese hypotrichosis simplex patients associated with LSS gene. *J. Dermatol.* 48(3), 408-412. doi:10.1111/1346-8138.15697

8 Yujing, Zhang., Mengxi, Zhao., Xiangqian, Li., Yongping, Zhao., Yijie, Sun., Jianzhong, Zhang., et al.(2025). Hypotrichosis 14: novel variants of the LSS gene in five Chinese families and insights from literature review. *Hum. Genomics* 19(1), 84. doi:10.1186/s40246-025-00798-7

9 Nicole, Cesarato., Maria, Wehner., Mariam, Ghughunishvili., Axel, Schmidt., Daisy, Axt., Holger, Thiele., et al.(2021). Four hypotrichosis families with mutations in the gene LSS presenting with and without neurodevelopmental phenotypes. *Am. J. Med. Genet. A.* 185(12), 3900-3904. doi:10.1002/ajmg.a.62438

10 Mami, Murata., Ryota, Hayashi., Yoshio, Kawakami., Shin, Morizane., and Yutaka, Shimomura.(2020). Two cases of severe congenital hypotrichosis caused by compound heterozygous mutations in the LSS gene. J Dermatol, 48(3), 392-396. doi:10.1111/1346-8138.15679

11 Bei, Zhao., Yisi, Tang., Wenjing, Chen., Huiying, Wan., Jiyun, Yang.,and Xuejun, Chen.(2023). A novel homozygous mutation in LSS gene possibly causes hypotrichosis simplex in two siblings of a Tibetan family from the western Sichuan province of China. *Front. Physiol.* 13(0), 992190. doi:10.3389/fphys.2022.992190

12 Nesma M, Elaraby., Hoda A, Ahmed., Neveen A, Ashaat., Sameh, Tawfik., Mahmoud K H, Ahmed., Nehal F, Hassib., et al.(2022). Expanding the Phenotypic Spectrum of APMR4 Syndrome Caused by a Novel Variant in LSS Gene and Review of Literature. *J. Mol. Neurosci.* 72(11), 2242-2251. doi:10.1007/s12031-022-02074-y

13 Hasnaa M, Elbendary., Dana, Marafi., Ahmed K, Saad., Rasha, Elhossini., Ruizhi, Duan., Karima, Rafat., Shalini N, Jhangiani., et al.(2023). Novel LSS variants in alopecia and intellectual disability syndrome: New case report and clinical spectrum of LSS-related rare disease traits. *Clin. Genet.* 104(3), 344-349. doi:10.1111/cge.14348

14 Ho, S., Lo, I. F. M., & Luk, H. M.(2022). Expansion of phenotype of lanosterol synthase-related disease: a case report and literature review. *hong kong journal of paediatrics* 27(1), 37-41.

15 Fang, Yang., Xingyuan, Jiang., Yuhao, Zhu., Mingyang, Lee., Zhengren, Xu., Jianglin, Zhang., et al.(2022). Biallelic Variants in Lanosterol Synthase (LSS) Cause Palmoplantar Keratoderma-Congenital Alopecia Syndrome Type 2. *J. Invest. Dermatol.* 142(10), 2687-2694.e2. doi:10.1016/j.jid.2022.03.023

16 Shengru, Zhou., Xingyuan, Jiang., Yuhao, Zhu., Jianqiu, Yang., Chunyu, Yuan., Min, Chen., et al.(2023). Biallelic mutations in LSS in autosomal-recessive mutilating palmoplantar keratoderma. *Exp. Dermatol.* 32(5), 699-706. doi:10.1111/exd.14774

17 Linlin, Bao., Qian, Li., Zhicao, Yue.,and Fang, Yang.(2025). Congenital hypotrichosis caused by compound heterozygous variants in the LSS gene in a Chinese patient with strabismus: case report. *Front. Pediatr.* 13(0), 1512646. doi:10.3389/fped.2025.1512646

18 Joelle, El Hakim., Cybel, Mehawej., Eliane, Chouery., Andre, Megarbane., Jinia, El-Feghaly., and Jinane, El Khoury.(2023). Non-syndromic hypotrichosis: A report of two novel variants in the LSS gene. *Pediatr. Dermatol.* 40(5), 960-961. doi:10.1111/pde.15320

19 Fanny, Morice-Picard., Pierre-Louis, Lanvin., Eulalie, Lasseaux., Franck, Boralevi., Christine, Léauté-Labrèze.,and Louis, Lebreton.(2023). A novel compound heterozygous variant in the LLS gene is associated with nonsyndromic hypotrichosis.*Clin. Exp. Dermatol.* 48(9), 1087-1089. doi:10.1093/ced/llad174
